# Business Strategy and Environmental Information Disclosure Quality: Empirical Evidence from Chinese Heavy Pollution Listed Firms

**DOI:** 10.3390/ijerph19148325

**Published:** 2022-07-07

**Authors:** Zhengguang Li, Ping Li, Xibo Zhao, Ziying Tu

**Affiliations:** 1 School of Economics and Management, Yancheng Institute of Technology, Yancheng 224051, China; lzgrm@ycit.edu.cn (Z.L.); tzyjg@ycit.edu.cn (Z.T.); 2 School of Management, Jiangsu University, Zhenjiang 212013, China; 3 School of Business, Renmin University of China, Beijing 100872, China

**Keywords:** environmental information disclosure quality, China, business strategy, heavy pollution industry, prospector-type firms, defender-type firms

## Abstract

Using the data of listed firms in China’s A-share heavy pollution industry between 2008 and 2020, based on organizational theory, this study examines the impact of prospector-type firms and defender-type firms on environmental information disclosure quality. Empirical evidence shows that prospector-type firms reduce environmental information disclosure quality, compared with defender-type firms. After a series of robustness tests, the conclusion is still valid. This paper tests the impact mechanism of business strategy on environmental information disclosure quality and finds that financing constraints play a mediating effect in the relationship between business strategy and environmental information disclosure quality. This paper enriches and expands the literature in the field of influencing factors of environmental information disclosure quality and economic consequences of business strategy. At the same time, the conclusion of this paper has important reference significance for regulators to formulate policies to improve environmental information disclosure quality according to the heterogeneity of business strategy.

## 1. Introduction

With the rapid development of the world economy, the environment is deteriorating [1], and the problem of global warming is becoming more and more serious. Global warming will become a major factor affecting human health in the future. The perplexity of extreme high temperature mainly reflecting the increase of incidence rate and mortality. The impact of global warming on human health has attracted great attention from all countries, including China. China has always actively participated in international governance and cooperation on global climate change. It has actively participated in the implementation negotiations and responsibility implementation since the signing of the 1992 United Nations Framework Convention on climate change, the 1997 Kyoto Protocol, the 2009 Copenhagen climate agreement and the 2015 Paris Agreement. In 2016, China played a historic role in promoting the conclusion, entry into force and formulation of implementation rules of the Paris Agreement. Marked by the implementation of the Paris Agreement, the Chinese government is more actively committed to the unification of global climate change response at the international and domestic levels. The 14th five-year plan for China’s national economic and social development proposes that carbon dioxide emissions should reach a peak by 2030 and strive to achieve carbon neutrality by 2060. China is taking action to achieve this goal. Remarkable results have been achieved. By the end of 2020, China’s carbon dioxide emissions per unit of GDP have decreased by 48.4% compared with 2005, exceeding the 40% to 45% target that China has promised to the international community.

As the main emitter of carbon dioxide, firms are the key subjects to achieve the “double carbon” goal. The Chinese government has always believed that high-quality environmental information disclosure is not only an important channel for the government to promote the modern environmental governance system, but also an important basis for reducing global warming. It has successively issued a number of policies to regulate environmental information disclosure. Since 2003, the Chinese government has revised the management measures for firm environmental information disclosure four times. After continuous revision, the measures for firm environmental information disclosure have been gradually improved and the supervision has been gradually strengthened. The newly revised firm environmental information disclosure management measures play an important role in supervising firms in fully disclosing environmental information in a timely manner, fulfilling environmental protection responsibilities, reducing emissions of harmful gases such as carbon dioxide and mitigating global warming. It can be seen that it is of great significance to find out what factors affect environmental information disclosure quality and put forward policy recommendations on this basis to actively respond to global warming and protect human health. As the overall and long-term planning of firms, business strategy is a series of agreements and actions carried out by a firm in order to develop its core competitiveness and obtain a competitive advantage. Business strategy is the starting point and basis of a series of firm decisions [2]. The strategy choice of firms will have a direct impact on the various behaviors of firms, of course including the impact on the behavior of environmental information disclosure. However, little literature examines the influencing factors of environmental information disclosure quality from the perspective of business strategy.

Business strategy is formed in the early stage of the firm life cycle and remains stable for a long time. As a comprehensive feature of firms, in theory, business strategy will have an important impact on the firm’s decisions and behaviors [3]. However, it was not until Bentley et al. (2013) constructed the corresponding scoring method according to typologies of business strategy proposed by Miles and Snow (1978, 2003) that academics begin to gradually focus on research in the field of economic consequences of business strategy [4,5,6]. However, there is only a limited amount of literature examining the economic consequences of business strategy from the perspective of environmental information disclosure quality. Therefore, this paper examines the economic consequences of business strategy from the perspective of environmental information disclosure quality, which is of great significance to broaden the research boundary in the field of economic consequences of business strategy.

To sum up, this paper examines the influencing factors of environmental information disclosure quality from the perspective of business strategy. On this basis, according to the relationship between business strategy and environmental information disclosure quality, this paper puts forward countermeasures to improve environmental information disclosure quality, so as to curb global warming and promote human health.

In order to solve the above problems, based on organizational theory proposed by Miles and Snow (1978, 2003), business strategy is divided into defenders, analyzers and prospectors [5,6]. Prospector-type firms are innovation oriented, focusing on developing new products and looking for new market opportunities. Defender-type firms refer to firms that focus on efficiency when producing products and providing services. The market situation of defender-type firms is generally narrow. Defender-type firms are often limited to a small number of specific types of products and services and rarely develop new products and markets. Compared with prospector-type firms, defender-type firms usually grow slowly and stably. Further, we use the business strategy classification construction scoring method proposed by Bentley et al. (2013) to measure business strategy [4]. Using the data of listed firms in China’s A-share heavy pollution industry between 2008 and 2020, this study examines the impact of prospector-type firms and defender-type firms on environmental information disclosure quality. Empirical evidence shows that prospector-type firms reduce environmental information disclosure quality, compared with defender-type firms. After a series of robustness tests, the conclusion is still valid. This paper tests the impact mechanism of business strategy on environmental information disclosure quality and finds that financing constraints play an intermediary role in the relationship between business strategy and environmental information disclosure quality.

This study has the following contributions: First, it enriches and expands the literature in the field of influencing factors of environmental information disclosure quality. The existing literature mainly examines the influencing factors of environmental information disclosure quality from the perspectives of government regulation (Darrell and Schwartz, 1997 [7]; Cho and Patten, 2007 [8]; Huang and Kung, 2010 [9]), corporate governance (Brammer and Pavelin, 2006 [10]; Zeng et al., 2012 [11]; Okere et al., 2021 [12], Gerged, 2021 [13]), firm characteristics (Brammer and Pavelin, 2008 [14]; Lu and Abeysekera, 2014 [15]; Cormier et al., 2005 [16]; Ismail et al., 2018 [17]; Kumar, 2021 [18]), executive characteristics (Lewis et al., 2014 [19]; Chen et al., 2021 [20]; Caputo et al., 2021 [21]), culture and institution (Buhr and Freedman, 2001) [22], media attention (Rupley et al., 2012 [23]; Solikhah and Maulina, 2021 [24]), political connection (Li et al., 2022 [25]), air quality (Wang et al., 2021 [26]), institutional ownership (Tarkhouni et al., 2020 [27], Li et al., 2022 [28]) and green credit (Zhan, 2021 [29]). This study examines the influencing factors of environmental information disclosure quality from the perspective of business strategy, so as to broaden the research boundary of the influencing factors of environmental information disclosure quality. Second, it enriches and expands the research in the field of economic consequences of business strategy. The existing literature mainly examines the economic consequences of business strategy from the perspectives of financial report fraud (Bentley et al., 2013 [4]), tax avoidance (Higgins et al., 2015 [30]), investment efficiency (Navissi et al., 2017 [31]), financial report readability (Lim et al., 2018 [32]; Habib and Hasan, 2020 [33]), audit fees (Bentley et al., 2013 [4]), stock price crash risk (Habib and Hasan, 2017 [34]), corporate social responsibility performance (Yuan et al., 2020 [35]), environmental inefficiency (Magerakis and Habib, 2021 [3]), sustainable development (Maniora, 2018 [36]; Liu and Kong, 2021 [37]) and internal control (Bentley-Goode et al., 2017 [38]). Taking environmental information disclosure quality as the starting point, this study examines the economic consequences of business strategy, so as to deepen the research on the impact of business strategy on firm decision-making and behavior. Third, the research conclusion of this study is helpful to provide reference for regulators to manage environmental information disclosure according to the heterogeneity of business strategy. This paper finds that compared with defender-type firms, prospector-type firms reduce environmental information disclosure quality, that is, the more aggressive the business strategy is, the worse the environmental information disclosure quality is. The conclusion of this study provides a reference for regulators to fully consider firm business strategy heterogeneity in the process of environmental information disclosure management, so as to curb global warming and promote human health.

The remainder of this paper is structured as follows: Section 2 reviews the literature and develops the hypothesis. Section 3 introduces the research design. Section 4 presents the empirical results. Section 5 is the robustness test. Section 6 makes additional analysis. Finally, Section 7 concludes the paper.

## 2. Literature Review and Hypothesis Development

### 2.1. Business Strategy

There are several typologies of business strategy in the literature of organization theory. These typologies describe how firms compete in their respective business fields. For example, Miles and Snow (1978 [5], 2003 [6]) classified business strategy into defenders, analyzers and prospectors. Porter (1980) [39] divided business strategy into cost leadership strategy and product differentiation strategy. March (1991) [40] divided typologies strategy into exploration strategy and utilization strategy. These typologies try to build their own strategy chains, so as to better classify different firms in reality.

In fact, the typologies of business strategy proposed by Miles and Snow (1978 [5], 2003 [6]) are similar to those proposed by Porter (1980) [39], March (1991) [40] (Dent, 1990 [41]; Langfield-Smith,1997 [42]). Prospectors proposed by Miles and Snow (1978 [5], 2003 [6]) integrates Porter’s product differentiation strategy and March’s exploration strategy. Defenders proposed by Miles and Snow (1978 [5], 2003 [6]) integrates Porter’s cost leadership strategy and March’s utilization strategy.

Compared with other typologies of business strategy, the typologies proposed by Miles and Snow (1978 [5], 2003 [6]) have the following advantages: First, their typologies of business strategy have been widely verified by a large number of effectiveness tests. Much existing literature supports the effectiveness of Miles and Snow (1978 [5], 2003 [6]) on typologies of business strategy in different scenarios. Second, their typologies of business strategy have strong and detailed theoretical guidance (Smith et al., 1989 [43]). Third, their typologies of business strategy are based on the in-depth analysis of various industries. The typologies of business strategy are universal in the context of various industries. Fourth, their typologies of business strategy can be divided by archival data (Ittner et al., 1997 [44]), while other typologies of business strategy can only be divided by survey data and interview data.

From the above analysis, it is not difficult to see that a large number of studies have confirmed that the typologies of business strategy proposed by Miles and Snow (1978 [5], 2003 [6]) are more scientific and reasonable and more applicable in various industries (Snow and Hambrick, 1980 [45]; Conant et al., 1990 [46]). Therefore, when examining the influencing factors of environment information disclosure quality from the perspective of business strategy heterogeneity, this paper also uses the typologies of business strategy proposed by Miles and Snow (1978 [5], 2003 [6]) for reference and divides business strategy into prospectors, defenders and analyzers. The specific characteristics of these three typologies of business strategy are as follows:

Prospector-type firms are innovation oriented, focusing on developing new products and looking for new market opportunities. Prospector-type firms focus on research and development and market development. Because they mainly rely on new products and new markets to promote growth, prospector-type firms grow rapidly and fluctuate violently. In terms of organization, due to the diversity of products, prospector-type firms usually have a large number of branches, which are highly dispersed and unstable, and pay more attention to the knowledge and skills of employees. Defender-type firms refer to firms that focus on efficiency when producing products and providing services. The market situation of defender-type firms is generally narrow. Defender-type firms are often limited to a small number of specific types of products and services and rarely develop new products and markets. Compared with prospector-type firms, defender-type firms usually grow slowly and stably. In terms of organization, defender-type firms usually have an efficient and centralized management and control system, employees have long tenures and internal promotion is common. Analyzer-type firms are between defender-type firms and prospector-type firms.

By analyzing the above characteristics of business strategy, we can find that compared with defender-type firms, prospector-type firms have greater R&D investment, stronger market development ability, more product types, more decentralized organizational structure, greater uncertainty in the future, worse profitability and future ability to earn cash flow and more serious financing constraints.

Because defender-type firms and prospector-type firms have different characteristics, a large amount of literature examines the impact of business strategy on corporate financial behavior from the perspective of business strategic heterogeneity. Research shows that compared with defender-type firms, prospector-type firms produce more immoral behaviors. For example, compared with defender-type firms, prospector-type firms have worse internal control (Bentley-Goode et al., 2017 [38]), higher degree of tax avoidance (Higgins et al., 2015 [30]), lower investment efficiency (Navissi et al., 2017 [31]), higher degree of financial report fraud (Bentley et al., 2013 [4]) and worse readability of financial reports (Lim et al., 2018 [32]; Habib and Hasan, 2020 [33]).

In addition, some literature finds that compared with defender-type firms, prospector-type firms have higher audit fees (Bentley et al., 2013 [4]), high risk of stock price crash (Habib and Hasan, 2017 [34]), lower sustainability(Maniora, 2018 [36]; Liu and Kong, 2021 [37]), higher corporate social responsibility performance (Yuan et al., 2020 [35]), higher environmental efficiency (Magerakis and Habib, 2021 [3]).

In Table 1 summary of main research conclusions on economic consequences of business strategy are presented.

### 2.2. Environmental Information Disclosure Quality

This paper examines the influencing factors of environmental information disclosure quality from the perspective of business strategy, so this part only reviews the literature in the field of influencing factors of environmental information disclosure quality. Table 2 presents a summary of main research conclusions on influencing factors of environmental information disclosure quality. As can be seen from Table 2, regulatory requirements of the government (Darrell and Schwartz, 1997 [7]; Cho and Patten, 2007 [8]; Huang and Kung, 2010 [9]), the pressure of public policy (Darrell and Schwartz, 1997 [7]; Cho and Patten, 2007 [8]; Huang and Kung, 2010 [9]), decentralized ownership structure of a company (Brammer and Pavelin, 2006 [10]), board independence (Okere et al., 2021 [12]; Gerged, 2021 [13]), “China famous brand” trademark (Zeng et al., 2012 [11]), firm size (Brammer and Pavelin, 2008 [14]; Lu and Abeysekera, 2014 [15]; Cormier et al., 2005 [16]; Ismail et al., 2018 [17]; Kumar, 2021 [18]), profitability (Brammer and Pavelin, 2008 [14]; Lu and Abeysekera, 2014 [15]; Cormier et al., 2005 [16]; Ismail et al., 2018 [17]; Kumar, 2021 [18]), newly appointed CEOs (Lewis et al., 2014 [19]), CEOs with MBA degrees (Lewis et al., 2014 [19]), a collectivist society (Buhr and Freedman, 2001 [22]), media report (Rupley et al., 2012 [23]; Solikhah and Maulina, 2021 [34]), institutional ownership (Tarkhouni et al., 2020 [27]; Li et al., 2022 [28]) and green credit (Zhan, 2021 [29]) positively correlated with environmental information disclosure quality. The separation of corporate control and cash flow rights (Zeng et al., 2012 [11]), financial leverage (Brammer and Pavelin, 2008 [14]; Lu and Abeysekera, 2014 [15]; Cormier et al., 2005 [16]; Ismail et al., 2018 [17]; Kumar, 2021 [18]), CEOs with a legal background (Lewis et al., 2014 [19]), executives’ military experience (Chen et al., 2021 [20]), CEO duality (Caputo et al., 2021 [21]), individualist society (Buhr and Freedman, 2001 [22]), highly politically connected executives (Li et al., 2022 [25]) and air quality (Wang et al., 2021 [26]) negatively correlated with environmental information disclosure quality.

Although there is a lot of literature examining the influencing factors of environmental information disclosure quality from different perspectives, this literature ignores the important factor of business strategy, which induces the research motivation of this paper.

### 2.3. Hypothesis Development

Business strategy determines the overall development direction of the firm in the future. The implementation of the business strategy directly affects the decision-making and behavior of the firm, of course, including the impact on the environmental information disclosure behavior of the firm. Because business strategy can be classified according to different standards, we speculate that different types of business strategy have different effects on environmental information disclosure quality. Therefore, when discussing the relationship between business strategy and environmental information disclosure quality, we need to consider the impact of business strategy heterogeneity.

Based on typologies of business strategy proposed by Miles and Snow (1978 [5], 2003 [6]), this study divides business strategy into defenders, analyzers and prospectors. These typologies of business strategy may exist in different enterprises in the same industry at the same time. Different typologies of business strategy have different characteristics. Defender-type firms follow efficiency-oriented strategy. Defender-type firms pay attention to cost leadership, focus on production and sales in narrow market areas, have less R&D investment, less product types, less employee changes, have a stable organizational structure, face less uncertainty, have strong profitability and ability to earn cash flow in the future and have low financing constraints. Generally, immoral behavior will not occur. Different from defender-type firms, prospector-type firms follow innovation-oriented strategy. Prospector-type firms focus on R&D investment, constantly opening up new markets and launching new products. There are many kinds of products, decentralized organizational structure, poor stability of organizational structure, frequent changes of employees, more R&D investment and growth opportunities, greater uncertainty, poor profitability and future cash flow earning ability and high degree of financing constraints, and they are more prone to immoral behavior. Analyzer-type firms have the characteristics of both prospector-type firms and defender-type firms. On the one hand, they pursue efficiency; on the other hand, they pay attention to R&D innovation. The existing literature mainly takes the analyzers as the benchmark and examines the impact of defenders and prospectors on firm decision-making and behavior (Bentley et al., 2013 [4]; Higgins et al., 2015 [30]; Bentley-Goode et al., 2017 [38]; Habib and Hasan, 2017 [34]; Lim et al., 2018 [32]; Habib and Hasan, 2020 [33]).

According to the different characteristics of defenders and prospectors, we expect that compared with defender-type firms, environmental information disclosure quality of prospector-type firms is worse, which can be explained in two aspects.

On the one hand, compared with defender-type firms, prospector-type firms face innovative investment activities such as new product R&D. Although prospector-type firms have strong growth, high uncertainty and poor profitability and ability to obtain cash flow in the future, they often have difficulty obtaining the necessary financial support for R&D investment. They are faced with large capital demand and high financing constraints. With a higher the degree of financing constraints, prospector-type firms will reduce environmental protection expenditure, fail to fulfill environmental protection obligations and have poor environmental protection performance. Existing literature studies have shown that there is a positive correlation between firm environmental protection performance and environmental information disclosure quality (Clarkson et al., 2008 [47]). That is, the worse the environmental performance of a firm, the worse the environmental information disclosure quality. Thus, compared with defender-type firms, prospector-type firms have lower environmental information disclosure quality.

On the other hand, compared with defender-type firms, prospector-type firms will have more immoral behaviors. For example, Higgins et al. (2015) [30] find that compared with defender-type firms, prospector-type firms have a higher degree of tax avoidance. Bentley et al. (2013) [4] find that compared with defender-type firms, prospector-type firms have higher financial reports fraud. Lim et al. (2018) [32] and Habib and Hasan (2020) [33] find that compared with defender-type firms, the readability of financial reports of prospector-type firms is worse. Liu and Kong (2021) [37] use green innovation as the proxy variable of sustainable development and investigate the impact of business strategy on sustainable development. They find that compared with defender-type firms, prospector-type firms engage in less sustainable development behavior. As a market participant, firms should take environmental protection as their own responsibility in the process of pursuing economic benefits. However, different strategic types of firms have differences in fulfilling the social responsibility of environmental protection obligations. According to the research conclusion that prospector-type firms are more prone to unethical behavior found in the existing literature, it is not difficult to assume that, compared with defender-type firms, prospector-type firms will also behave unethically with regards environmental protection. That is, the less environmental protection responsibilities and obligations are fulfilled, the worse environmental protection performance and environmental information disclosure quality is.

To sum up, we develop the following hypothesis.

**Hypothesis** **1** **(H1).** *Ceteris paribus, prospector-type firms have lower environmental information disclosure quality than defender-type firms*.

## 3. Research Design

### 3.1. Sample Selection and Data Source

This paper selects the listed firms with industry codes B, C and D in China from 2008 to 2020 as the research object. After excluding the samples of ST, *St and missing data, this paper obtains 10,421 samples, the sample size of industry B, C and D are 383, 9531 and 507, respectively. In order to mitigate the possible impact of outliers on the research conclusion, we winsorize the tail of all continuous variables by 1% up and down.

### 3.2. Measuring Business Strategy

Based on the research of Bentley et al. (2013) [4], this study constructs a discrete variable to measure business strategy. This variable focuses on the following six characteristics of the firm.

(1) The propensity of the firm to look for new products. It is measured by the average ratio of R&D expenditure to sales in the past five years. Compared with defender-type firms, prospector-type firms usually have more innovative behavior, so prospector-type firms will have more R&D expenditure (Hambrick, 1983 [48]).
(2) The efficiency of firms in producing, selling products and providing services. It is measured by the average ratio of the number of employees to sales in the past five years. This indicator measures the firm’s ability to produce and deliver products and services. Compared with defender-type firms, prospector-type firms have lower requirements for efficiency, so they need more personnel per unit of sale (Thomas et al., 1991 [49]).
(3) Historical growth rate of the firm. It is measured by the average growth rate of sales in the past five years. Compared with defender-type firms, prospector-type firms usually have stronger growth ability.
(4) The attention of the firm on developing new products and services. It is measured by the average ratio of the sum of sales expenses and management expenses to sales in the past five years. Compared with defender-type firms, prospector-type firms usually have higher sales expenses and management expenses, so as to expand the product market (Hambrick, 1983 [48]).
(5) Organization stability. It is measured by the standard deviation of total employees in the past five years. Compared with defender-type firms, prospector-type firms usually have weaker organizational stability and shorter employee tenure (Miles and Snow, 1978 [5], 2003 [6]).
(6) Capital intensity. It is measured by the average ratio of net property, plant and equipment to total assets in the past five years. Prospector-type firms usually have high human resource density, while defender-type firms usually have high capital density (Hambrick, 1983 [48]).

Building on the research of Bentley et al. (2013) [3], all variables of business strategy are computed over a rolling prior five-year average. We rank these variables by forming quintiles within each industry-year. For the first five dimensions, all firm-year observations are given scores of 5, 4, 3, 2 or 1 in turn from high to low quintile. However, the last dimension is opposite; all firm-year observations are given scores of 1, 2, 3, 4 or 5 in turn from high to low quintile.

Then, we compute the total scores across the six dimensions for each firm-year such that the maximum strategy is 30 (prospectors) and the minimum strategy score is 6 (defenders). High strategy scores represent prospector-type firms, whereas low strategy scores represent defender-type firms (Bentley et al., 2013 [4]). According to the methods of Bentley et al. (2013) [4] and Higgins et al. (2015) [30], a score of 6–12 indicates defender-type firms, a score of 24–30 indicates prospector-type firms and a score of 13–23 indicates analyzer-type firms. Referring to the research of Bentley et al. (2013) [4], this paper uses the discrete score variable of business strategy for regression in empirical analysis. The larger the score, the more aggressive the business strategy is; the smaller the score, the more defensive the business strategy is.

### 3.3. Measuring Environmental Information Disclosure Quality

In Table 3, the rural ecological environment governance efficiency index system is presented. According to the assignment method (Wiseman, 1982 [50]; Li et al., 2022 [28]) in Table 3, we can calculate the total score of firm environmental information disclosure quality and then take natural logarithm as the proxy variable of firm environmental information disclosure quality (*Eidq*).

### 3.4. Model Specification

Since this study assumes that there is a linear relationship between business strategy and environmental information disclosure quality, this paper uses the method of ordinary least squares (OLS) to test this hypothesis. Based on the research of Clarkson et al. (2008) [47], Zeng et al. (2012) [11], Lu and Abeysekera (2014) [15], Ismail et al. (2018) [17] and Fan et al. (2020) [51], this study uses the following Model (1) to test the impact of business strategy on environmental information disclosure quality:

(1) $$\begin{array}{cc} Ediq & =\beta_{0}+\beta_{1}Strategy+\beta_{2}Roa+\beta_{3}Growth+\beta_{4}Lev+\\ & \beta_{5}Size+\beta_{6}First+\beta_{7}Board+\beta_{8}Ddbl+\beta_{9}Dual+\varepsilon \end{array}$$

See Table 3 for specific definitions of variables. In Model (1), we only focus on $\beta_{1}$, if $\beta_{1}$ is significantly negative, H1 is verified.

## 4. Empirical Results

### 4.1. Descriptive Statistics

Table 4 the descriptive statistical of the main variables are presented. The mean (median) of environmental information disclosure quality (*Eidq*) is 2.104 (2.197), and the standard deviation is 0.918, which indicates that there is no significant difference in environmental information disclosure quality (*Eidq*) within the sample. The mean (median) of business strategy (*Strategy*) is 17.905 (18), and Q3 is 21, which indicates that more than 75% of the samples are defender-type firms and analyzer-type firms, and the standard deviation is 4.166, which indicates that there is great difference in business strategy (*Strategy*) within the sample.

### 4.2. Correlation Matrix

Table 5 shows the correlation matrix for variables used in the study. The lower(upper) left(right) triangle reports the Pearson (Spearman) correlations. The Pearson (Spearman) correlation coefficient shows a significant and negative relationship between *Strategy* and *Eidq*, which indicates that the univariate analysis supports H1. The correlation coefficients between other variables are below 0.5, indicating that there is no serious multicollinearity. In order to control the impact of other factors on environmental information disclosure quality, we conduct multiple regression analysis below.

### 4.3. Multiple Regression Results

In Table 6, the multiple regression results of the impact of business strategy on environmental information disclosure quality are presented. In column 1 of Table 6, the multiple regression results of the impact of business strategy on environmental information disclosure quality without controlling other variables are presented. In column 2 of Table 6, the multiple regression results of the impact of business strategy on environmental information disclosure quality under control of other variables are presented.

Column 1 of Table 6 shows that there is a significantly negative correlation between *Strategy* and *Eidq* ($\beta_{1}$ = −0.036, t = −16.80), which indicates that prospector-type firms have lower environmental information disclosure quality than defender-type firms; H1 of the study is verified. Column 2 of Table 6 shows that there is a significantly negative correlation between *Strategy* and *Eidq* ($\beta_{1}$ = −0.033, t = −16.62), which indicates that prospector-type firms have lower environmental information disclosure quality than defender-type firms; H1 of the study is verified.

In terms of control variables, the impact coefficient of *Roa* is 0.455, and it is significant at the 1% level, the impact coefficient of *Growth* is −0.132, and it is significant at the 1% level, the impact coefficient of *Lev* is −0.449, and it is significant at the 1% level, the impact coefficient of *Size* is 0.289, and it is significant at the 1% level, the impact coefficient of *First* is 0.464, and it is significant at the 1% level and the impact coefficient of *Board* is 0.383, and it is significant at the 1% level. These research conclusions are basically consistent with the conclusions of the existing literature (Lu and Abeysekera, 2014 [15]; Ismail et al., 2018 [17]; Fan et al., 2020 [51]; Clarkson et al., 2007 [47]; Zeng et al., 2012 [11]). The impact coefficient of *Ddbl* is 0.327, and it is significant at the 10% level, and the impact coefficient of *Dual* is −0.045, and it is significant at the 5% level.

## 5. Robust Tests

### 5.1. Endogeneity Tests

So far, our analysis provides robust evidence that the environmental information disclosure quality of prospector-type firms is worse than defender-type firms. However, there may be a concern that our conclusion may also have reverse causality. That is, due to the worse environmental information disclosure quality, the more aggressive the business strategy. We adopt the following two methods to mitigate this concern: (1) the business strategy of one lag period to measure business strategy (*LagStrategy*) is adopted; (2) the industry-year average of business strategy is used as the instrumental variable (*Strategy_Mean*). We believe that this instrumental variable meets the requirements of correlation and exogeneity. In terms of relevance, firms in the same industry faced similar industry characteristics and external environment in the same year, so there is a certain correlation between their business strategies. At present, there is no evidence that the business strategy of other firms in the same industry in the same year will affect the environmental information disclosure quality of the firm, so it meets the exogenous principle.

Table 7 shows the endogeneity test results. In column 1 of Table 7, the multiple regression results of the impact of the business strategy of one lag period on environmental information disclosure quality are presented. We can find that there is a significantly negative correlation between *LagStrategy* and *Eidq*, and H1 of the study is verified again. In columns 2 and 3 of Table 7, the multiple regression results of the impact of business strategy on the environmental information disclosure quality when industry-year average of business strategy is used as the instrumental variable are presented. From columns 2 and 3 of Table 7, we can find that there is still a significantly negative correlation between *Strategy_IV* and *Eidq*, and H1 is verified again.

### 5.2. Alternative Measures of Business Strategy and Environmental Information Disclosure Quality

In order to test the sensitivity of the research conclusion to the business strategy measurement, we use the following two indicators as alternative business strategy measures: (1) *Strategy* in the Model (1) is replaced by *Pros* representing prospector-type firms and *Defe* representing defender-type firms. Following Bentley et al. (2013) [4], when *Strategy* ≥ 24, the value of *Pros* is 1, otherwise, it is 0; when *Strategy* ≤ 12, the value of *Defe* value is 1, otherwise, it is 0. (2) Following Magerakis and Habib (2021) [3], we use the natural logarithm of business strategy (*LnStratrgy*) as an alternative business strategy measure.

In order to test the sensitivity of the research conclusion to environmental information disclosure quality, we also use the environmental information disclosure quality divided by its possible maximum value of 50 as an alternative environmental information disclosure quality measure (*Eidq/Max*).

Table 8 shows the regression results. From columns 1 and 2 of Table 8, we can find that the two newly defined business strategy measurement indicators (*Pros*) are still significantly negatively correlated with the environmental information disclosure quality, which is in line with the expectation of H1, indicating that the research conclusion of this paper is not sensitive to business strategy measurement indicators. From column 3 of Table 8, we can find that business strategy is significantly negatively correlated with newly defined environmental information disclosure quality (*Eidq/Max*), which is in line with the expectation of H1, which shows that the research conclusion of this paper is not sensitive to the environmental information disclosure quality measurement.

### 5.3. Groups Test of Firm Size

Following Bentley et al. (2013) [4], we believe that firm size may be the influencing factor of business strategy, so it is possible that the research conclusion of this paper is caused by firm size (the natural logarithm of the firm’s total assets at the end of the year). Therefore, this paper divides the samples into three groups from small to large according to the firm size of one lag period by industry-year and generates dummy variables of firm size: small firm (*SizeQ1*, small firm 1, otherwise 0), medium-sized firm (*SizeQ2*, medium-sized firm 1, otherwise 0) and large firm (*SizeQ3*, large firm 1, otherwise 0). Then, multiply strategy with *SizeQ1*, *SizeQ2* and *SizeQ3* to generate the interactive item *Strategy × SizeQ1*, *Strategy × SizeQ2* and *Strategy × SizeQ3*, which is brought into Model (1) of this paper for testing. In column 1 of Table 9, the research results are shown. From column 1 of Table 9, we find that strategy is still significantly negatively correlated with *Eidq*, but the coefficients of interaction terms *Strategy × SizeQ1*, *Strategy × SizeQ2* and *Strategy × SizeQ3* are not significant.

We also test H1 in three subsamples of small firm, medium-sized firm and large firm, respectively. In columns 2, 3 and 4 of Table 9, the research results are shown. We can find that in the three subsamples, the coefficient of *Strategy* is significantly negatively correlated at the level of 1%, which indicates that firm size does not affect the result in Table 6.

### 5.4. Panel Data

In order to solve the possible problems of heteroscedasticity, we use the fixed effect panel data to test the robustness of the research hypothesis in this paper. Table 10 the research results are shown, and the research conclusions have not changed substantially.

## 6. Additional Analysis

As mentioned above, business strategy will affect financing constraints, which will affect the environmental protection responsibilities performance and then affect the environmental information disclosure quality. In view of this, we further analyze the internal mechanism of business strategy affecting environmental information disclosure quality, that is, whether business strategy affects environmental information disclosure quality through financing constraints. We design Model (2) and Model (3), together with Model (1) above, to test the relationship between business strategy, financing constraints and environmental information disclosure quality.

(2) $$\begin{array}{cc} Finance & =\alpha_{0}+\alpha_{1}Strategy+\alpha_{2}Roa+\alpha_{3}Growth+\alpha_{4}Lev+\\ & \alpha_{5}Size+\alpha_{6}First+\alpha_{7}Board+\alpha_{8}Ddbl+\alpha_{9}Dual+\varepsilon \end{array}$$

(3) $$\begin{array}{cc} Eidq & =\gamma_{0}+\gamma_{1}Strategy+\gamma_{2}Finance+\gamma_{3}Roa+\gamma_{4}Growth+\gamma_{5}Lev+\\ & \gamma_{6}Size+\gamma_{7}First+\gamma_{8}Board+\gamma_{9}Ddbl+\gamma_{10}Dual+\varepsilon \end{array}$$

In Models (2) and (3), *Finance* represents financing constraints. We use the negative cash adequacy ratio to measure financing constraints. The larger the indicator, the greater the firm financing constraints. The definition of other variables is the same as that of Model (1).

The test results of the mediating effect of business strategy on environmental information disclosure quality through financing constraints are presented in Table 11. Column 1 of Table 11 shows that there is a significant negative correlation between *Strategy* and *Eidq*. Column 2 of Table 11 shows that there is a significant positive correlation between *Strategy* and *Finance*. Column 3 of Table 11 shows that there is a significant negative correlation between *Strategy* and *Eidq*, and there is a significant negative correlation between *Finance* and *Eidq*. Multiple regression results of columns 1, 2 and 3 in Table 11 show that the more aggressive the business strategy is, the higher the degree of financing constraint is, the higher the degree of financing constraint is, and the worse the environmental information disclosure quality is; financing constraints play a mediating effect in the relationship between business strategy and the environmental information disclosure quality. The effect size = −0.00049; according to Sobel (1982) [52], the calculation formula of the Z-test of the mediating path intensity $Z=\frac{\alpha_{1}\times \gamma_{2}}{\sqrt[{2}]{\gamma_{2}{}^{2}s^{2}{}_{\alpha_{1}}+\alpha_{1}{}^{2}s^{2}{}_{\gamma_{2}}}}$ = −2.97, and the *Z* value is mainly used to test whether the mediating effect is significant. The *Z* value in this paper is less than −2.5647, so the mediating effect is significant at the level of 1%, which indicates that financing constraints play a mediating role in the process of business strategy affecting environmental information disclosure quality, and the proportion of mediating effect in the total effect $\frac{\alpha_{1}\times \gamma_{2}}{\beta_{1}}$ = 1.59%.

## 7. Conclusions

The research results of this paper are based on the full disclosure of environmental information of the heavy pollution industry. The business environment of the heavy pollution industry has received strict supervision from the Chinese government. The Chinese government requires the heavy pollution industry to disclose environmental information compulsorily, which provides a data basis for us to obtain sufficient environmental information disclosure data to study.

Using the data of listed firms in China’s A-share heavy pollution industry between 2008 and 2020, based on Miles and Snow’s (1978 [4], 2003 [5]) organizational theory, this study examines the impact of prospector-type firms and defender-type firms on environmental information disclosure quality. Empirical evidence shows that prospector-type firms reduce environmental information disclosure quality, compared with defender-type firms. After a series of robustness tests, the conclusion is still valid. This paper tests the impact mechanism of business strategy on environmental information disclosure quality and finds that financing constraints play a mediating effect in the relationship between business strategy and environmental information disclosure quality. This paper enriches and expands the literature in the field of influencing factors of environmental information disclosure quality and economic consequences of business strategy. At the same time, the conclusion of this paper has important reference significance for regulators to formulate policies to improve environmental information disclosure quality according to the heterogeneity of business strategy.

The research conclusions of the study have important theoretical and practical significance. First, this study finds that business strategy, a comprehensive characteristic of firms, is an important variable affecting the environmental information disclosure quality, which breaks through the previous research on analyzing the environmental information disclosure quality only from the perspective of a single characteristic of the firm. Second, the research conclusion of the study shows that we should comprehensively and objectively understand the risks brought by business strategy to the environmental information disclosure quality, especially compared with defender-type firms, as environmental information disclosure quality of prospector-type firms is worse. This requires regulators to further strengthen the supervision of prospector-type firms and guide prospector-type firms to enhance their awareness of fulfilling their environmental responsibility and disclosing environmental information to external investors in a timely, full and high-quality manner. Third, this study finds that business strategy affects environmental information disclosure quality through financing constraints. This provides a reference for firm stakeholders to accurately understand the impact mechanism of business strategy on environmental information disclosure quality.

## Figures and Tables

**Table 1:** Summary of main research conclusions on economic consequences of business strategy.

Perspectives	Authors	Conclusions
environmental inefficiency	Magerakis and Habib (2021) [3]	environmental inefficiency (−)
financial report fraud	Bentley et al. (2013) [4]	financial report fraud (+)
audit fees	Bentley et al. (2013) [4]	audit fees (+)
tax avoidance	Higgins et al. (2015 ) [30]	tax avoidance (+)
investment efficiency	Navissi et al. (2017) [31]	investment efficiency (−)
financial report readability	Lim et al. (2018) [32], Habib and Hasan (2020) [33]	financial report readability (−)
stock price crash risk	Habib and Hasan (2017) [34]	stock price crash risk (+)
Corporate social responsibility performance	Yuan et al. (2020) [35]	Corporate social responsibility performance (+)
sustainable development	Maniora (2018) [36], Liu and Kong (2021) [37]	sustainable development behaviors (−)
internal control	Bentley-Goode et al. (2017) [38]	internal control (−)

Note: “+” indicates that business strategy is positively correlated with variables; “−” indicates that business strategy is negatively correlated with variables.

**Table 2:** Summary of main research conclusions on influencing factors of environmental information disclosure quality.

Perspectives	Authors	Conclusions
Government regulation	Darrell and Schwartz (1997) [7], Cho and Patten (2007) [8], Huang and Kung (2010) [9]	regulatory requirements of the government (+), the pressure of public policy (+)
Corporate governance	Brammer and Pavelin (2006) [10]	decentralized ownership structure of a company (+)
	Zeng (et al., 2012) [11]	the separation of corporate control and cash flow rights (−)
	Okere et al. (2021) [12], Gerged (2021) [13]	board independence (+)
Organizational impression and reputation	Zeng et al. (2012) [11]	“China famous brand” trademark (+)
Firm characteristics	Brammer and Pavelin (2008) [14], Lu and Abeysekera (2014) [15], Cormier et al. (2005) [16], Ismail et al. (2018) [17], Kumar (2021) [18]	firm size (+), profitability (+), financial leverage (−)
Executive characteristics	Lewis et al. (2014) [19]	newly appointed CEOs (+), CEOs with MBA degrees (+), CEOs with legal background (−)
	Chen et al. (2021) [20]	executives’ military experience (−)
	Caputo et al. (2021) [21]	CEO duality (−)
Culture and institution	Buhr and Freedman (2001) [22]	a collectivist society (+), individualist society (−)
Media attention	Rupley et al. (2012) [23], Solikhah and Maulina (2021) [24]	media report (+)
Political connection	Li et al. (2022) [25]	highly politically connected executives (−)
Air quality	Wang et al. (2021) [26]	air quality (−)
Institutional ownership	Tarkhouni et al. (2020) [27], Li et al. (2022) [28]	institutional ownership (+)
Green credit	Zhan (2021) [29]	green credit (+)

Note: “+” indicates that variables are positively correlated with environmental information disclosure quality; “−” indicates that variables are negatively correlated with environmental information disclosure quality.

**Table 3:** The rural ecological environment governance efficiency index system.

Disclosure Type	Disclosure Items	Scoring Description
Environmental management disclosure	Environmental protection concept	Disclosure: 2 points None: 0 point
	Environmental goals	
	Environmental protection management system	
	Environmental protection education and training	
	Special action of environmental protection	
	Environmental time emergency mechanism	
	Environmental protection honors or awards	
	“Three simultaneities” system	
Environmental certification disclosure	Whether it has passed ISO14001 certification	Yes: 2 points No: 0 point
	Whether it has passed ISO9001 certification	
Environmental information disclosure carrier	Annual Report of Listed Companies	Disclosure: 2 points None: 0 point
	Social Responsibility Report	
	Environmental Report	
Environmental liabilities disclosure	Wastewater emissions	Quantitative and qualitative description: 2 points Qualitative only: 1 point None: 0 point
	COD emissions	
	SO_2_ emissions	
	CO_2_ emissions	
	Smoke and dust emissions	
	Industrial solid waste emissions	
Environmental performance and governance disclosure	Waste gas emission reduction and treatment	
	Wastewater emission reduction and treatment	
	Dust and smoke control	
	Utilization and disposal of solid waste	
	Control of noise, light pollution and radiation	
	Implementation of cleaner production	

**Table 4:** Descriptive statistics of main variables.

Variable	Observations	Mean	Std. Dev.	Min.	Q1	Median	Q3	Max.
*Eidq*	10,421	2.104	0.918	0	1.386	2.197	2.890	3.892
*Strategy*	10,421	17.905	4.166	6	15	18	21	30
*Roa*	10,421	0.032	0.071	−0.290	0.009	0.031	0.063	0.225
*Growth*	10,421	0.150	0.412	−0.549	−0.040	0.087	0.234	2.673
*Lev*	10,421	0.461	0.197	0.071	0.312	0.463	0.606	0.932
*Size*	10,421	22.464	1.200	19.959	21.640	22.334	23.195	25.796
*First*	10,421	0.264	0.166	0.010	0.121	0.250	0.384	0.692
*Board*	10,421	2.153	0.200	1.609	2.079	2.197	2.197	2.708
*Ddbl*	10,421	0.373	0.053	0.313	0.333	0.333	0.417	0.571
*Dual*	10,421	0.213	0.410	0	0	0	0	1

**Table 5:** Correlation matrix (Pearson bottom and Spearman top).

Variable	*Eidq*	*Strategy*	*Roa*	*Growth*	*Lev*	*Size*	*First*	*Board*	*Ddbl*	*Dual*
*Eidq*	1	−0.167 ***	0.087 ***	−0.014	0.082 ***	0.382 ***	0.203 ***	0.171 ***	−0.021 **	−0.097 ***
*Strategy*	−0.162 ***	1	0.083 ***	0.105 ***	−0.090 ***	−0.014	−0.154 ***	−0.053 ***	0.019 **	0.104 ***
*Roa*	0.105 ***	0.028 ***	1	0.357 ***	−0.367 ***	0.097 ***	0.018 *	0.035 ***	−0.055 ***	0.019 *
*Growth*	−0.043 ***	0.066 ***	0.262 ***	1	−0.016 *	0.098 ***	−0.087 ***	0.009	−0.015	0.034 ***
*Lev*	0.070 ***	−0.075 ***	−0.340 ***	0.004	1	0.404 ***	0.104 ***	0.154 ***	−0.020 **	−0.083 ***
*Size*	0.385 ***	−0.004	0.123 ***	0.074 ***	0.383 ***	1	0.192 ***	0.229 ***	−0.009	−0.112 ***
*First*	0.199 ***	−0.140 ***	0.060 ***	−0.119 ***	0.103 ***	0.226 ***	1	0.069 ***	0.007	−0.153 ***
*Board*	0.176 ***	−0.042 ***	0.069 ***	−0.001	0.152 ***	0.260 ***	0.074 ***	1	−0.498 ***	−0.194***
*Ddbl*	−0.026 ***	0.014	−0.054 ***	−0.023 **	−0.015	−0.006	0.007	−0.487 ***	1	0.100 ***
*Dual*	−0.095 ***	0.100 ***	−0.005	0.025 **	−0.081 ***	−0.105 ***	−0.152 ***	−0.185 ***	0.105 ***	1

Note: lower(upper) left(right) triangle is Pearson (Spearman) correlations; *, ** and *** represent significance at 10, 5 and 1 percent, respectively (two-tailed).

**Table 6:** Business strategy and environmental information disclosure quality.

Variable	(1)	(2)
*Intercept*	2.745 ***	−4.662 ***
	(70.09)	(−23.46)
*Strategy*	−0.036 ***	−0.033 ***
	(−16.80)	(−16.62)
*Roa*		0.455 ***
		(3.45)
*Growth*		−0.132 ***
		(−6.38)
*Lev*		−0.449 ***
		(−9.09)
*Size*		0.289 ***
		(36.48)
*First*		0.464 ***
		(9.06)
*Board*		0.383 ***
		(7.84)
*Ddbl*		0.327 *
		(1.87)
*Dual*		−0.045 **
		(−2.23)
*Observations*	10,421	10421
*Adj. R* ^2^	0.026	0.201

Note: *t*-statistics are in parentheses; *, ** and *** indicate significance at the 10, 5 and 1 percent level, respectively.

**Table 7:** Business strategy and environmental information disclosure quality: Endogeneity tests.

Variable	(1) *Eidq*	(2) *Strategy* First Stage Regression	(3) *Eidq* Second Stage Regression
*Intercept*	−4.243 ***	2.544	−2.584 ***
	(−18.89)	(0.62)	(−2.69)
*LagStrategy*	−0.031 ***		
	(−14.11)		
*Strategy_Mean*		0.730 ***	
		(3.23)	
*Strategy_IV*			−0.167 ***
			(−2.75)
*Roa*	0.546 ***	−0.399	0.371 ***
	(3.69)	(−0.74)	(2.67)
*Growth*	−0.125 ***	0.028	−0.134 ***
	(−5.06)	(1.39)	(−6.26)
*Lev*	−0.387 ***	−1.495 ***	−0.659 ***
	(−6.78)	(−6.33)	(−6.04)
*Size*	0.267 ***	0.217 ***	0.320 ***
	(29.60)	(5.71)	(19.81)
*First*	0.564 ***	−3.332 ***	0.018
	(9.64)	(−13.36)	(0.09)
*Board*	0.430 ***	−0.479 **	0.315 ***
	(7.78)	(−1.98)	(5.39)
*Ddbl*	0.221	−0.367	0.276
	(1.13)	(−0.44)	(1.55)
*Dual*	−0.032	0.764 ***	0.058
	(−1.38)	(7.57)	(1.14)
*Observations*	7905	10421	10421
*Adj. R* ^2^	0.197	0.033	0.181

Note: *t*-statistics are in parentheses; ** and *** indicate significance at the 5 and 1 percent level, respectively.

**Table 8:** Business strategy and environmental information disclosure quality: Measurement index sensitivity test.

Variable	(1) *Eidq*	(2) *Eidq*	(3) *Eidq/Max*
*Intercept*	−5.282 ***	−3.740 ***	−1.227 ***
	(−26.76)	(−17.37)	(−30.44)
*Pros*	−0.295 ***		
	(−10.25)		
*Defe*	0.219 ***		
	(8.30)		
*Ln* *Strategy*		−0.526 ***	
		(−16.14)	
*Strategy*			−0.007 ***
			(−16.75)
*Roa*	0.460 ***	0.467 ***	0.042
	(3.48)	(3.54)	(1.58)
*Growth*	−0.144 ***	−0.133 ***	−0.022 ***
	(−6.94)	(−6.44)	(−5.26)
*Lev*	−0.425 ***	−0.440 ***	−0.094 ***
	(−8.58)	(−8.91)	(−9.36)
*Size*	0.290 ***	0.288 ***	0.063 ***
	(36.30)	(36.34)	(39.30)
*First*	0.501 ***	0.468 ***	0.104 ***
	(9.77)	(9.12)	(10.01)
*Board*	0.387 ***	0.388 ***	0.075 ***
	(7.88)	(7.93)	(7.53)
*Ddbl*	0.346 **	0.338 *	0.056
	(1.97)	(1.93)	(1.58)
*Dual*	−0.054 ***	−0.047 **	−0.009 **
	(−2.67)	(−2.31)	(−2.14)
*Observations*	10421	10421	10421
*Adj. R* ^2^	0.195	0.200	0.218

Note: *t*-statistics are in parentheses; *, ** and *** indicate significance at the 10, 5 and 1 percent level, respectively.

**Table 9:** Business strategy and environmental information disclosure quality: Groups test of firm size.

Variable	(1) *Eidq*	(2) *SizeQ1*	(3) *SizeQ2*	(4) *SizeQ3*
*Intercept*	1.101 *** (7.00)	1.397 *** (4.67)	1.527 *** (5.08)	0.971 *** (4.06)
*Strategy*	−0.062 * (−1.70)	−0.035 *** (−9.55)	−0.030 *** (−8.43)	−0.030 *** (−8.50)
*Strategy × SizeQ1*	0.017 (0.45)			
*Strategy × SizeQ2*	0.029 (0.79)			
*Strategy × SizeQ3*	0.049 (1.34)			
*Roa*	0.785 *** (5.79)	0.561 *** (2.84)	0.934 *** (3.73)	0.806 *** (2.82)
*Growth*	−0.125 *** (−5.88)	−0.050 (−1.25)	−0.166 *** (−4.37)	−0.146 *** (−4.39)
*Lev*	−0.223 *** (−4.43)	−0.217 *** (−2.83)	−0.274 *** (−2.97)	−0.282 *** (−2.79)
*First*	0.654 *** (12.54)	0.373 *** (3.84)	0.665 *** (7.18)	0.794 *** (9.62)
*Board*	0.561 *** (11.30)	0.423 *** (4.54)	0.387 *** (4.00)	0.708 *** (9.61)
*Ddbl*	0.754 *** (4.21)	0.364 (1.10)	0.527 (1.58)	0.989 *** (3.56)
*Dual*	−0.049 ** (−2.35)	−0.092 *** (−2.77)	−0.112 *** (−3.04)	0.083 ** (2.14)
*Observations*	10421	3462	3488	3471
*Adj. R* ^2^	0.155	0.052	0.066	0.094

Note: *t*-statistics are in parentheses; *, ** and *** indicate significance at the 10, 5 and 1 percent level, respectively.

**Table 10:** Business strategy and environmental information disclosure quality: Panel data.

Variable	(1)	(2)
*Intercept*	3.755 ***	−4.752 ***
	(9.20)	(−8.95)
*Strategy*	−0.019 ***	−0.023 ***
	(−6.25)	(−7.77)
*Roa*		−0.450 ***
		(−3.74)
*Growth*		−0.084 ***
		(−5.54)
*Lev*		−0.371 **
		(−5.69)
*Size*		0.349 ***
		(27.90)
*First*		0.281 ***
		(4.26)
*Board*		−0.116 *
		(−1.68)
*Ddbl*		0.441 **
		(2.14)
*Dual*		−0.019
		(−0.84)
*Observations*	10421	10421
*R* ^2^	0.684	0.712

Note: *t*-statistics are in parentheses; *, ** and *** indicate significance at the 10, 5 and 1 percent level, respectively.

**Table 11:** Business strategy, financing constraints and environmental information disclosure quality.

Variable	(1) *Eidq*	(2) *Finance*	(3) *Eidq*
*Intercept*	−4.699 ***	−1.067 **	−4.714 ***
	(−23.66)	(−2.32)	(−23.74)
*Strategy*	−0.032 ***	0.038 ***	−0.032 ***
	(−16.44)	(8.37)	(−16.13)
*Finance*			−0.013 ***
			(−3.17)
*Roa*	0.465 ***	−4.149 ***	0.409 ***
	(3.47)	(−13.41)	(3.03)
*Growth*	−0.133 ***	0.172 ***	−0.131 ***
	(−6.42)	(3.57)	(−6.31)
*Lev*	−0.447 ***	1.199 ***	−0.431 ***
	(−8.97)	(10.39)	(−8.61)
*Size*	0.290 ***	−0.066 ***	0.290 ***
	(36.38)	(−3.60)	(36.27)
*First*	0.465 ***	−0.220 *	0.462 ***
	(9.04)	(−1.85)	(8.98)
*Board*	0.385 ***	0.253 ***	0.389 ***
	(7.87)	(2.24)	(7.94)
*Ddbl*	0.342 **	0.282	0.346 **
	(2.03)	(0.72)	(2.06)
*Dual*	−0.044 **	0.026	−0.044 **
	(−2.17)	(0.55)	(−2.15)
*Observations*	10338	10338	10338
*Adj. R* ^2^	0.201	0.053	0.201

Note: *t*-statistics are in parentheses; *, ** and *** indicate significance at the 10, 5 and 1 percent level, respectively.

## Data Availability

No new data were created or analyzed in this study. Data sharing is not applicable to this article.
